# Durability of mitral valve reconstruction using the cosgrove edwards annuloplasty band at 5 years

**DOI:** 10.1186/1749-8090-8-S1-O290

**Published:** 2013-09-11

**Authors:** P Risteski, E von Spreti, F Sipahi, U Stock, M Doss, A Moritz, A Zierer

**Affiliations:** 1 Department of Thoracic and Cardiovascular Surgery, Johann Wolfgang Goethe University, Frankfurt am Main, Germany

## Background

In the past, questions have been raised, whether an open flexible annuloplasty band can reliably prevent recurrent mitral valve regurgitation. The purpose of this study was to evaluate the durability of mitral valve repair at midterm, using the Cosgrove-Edwards annuloplasty band in a homogenic patient cohort.

## Methods

From January 2004 to December 2007, 157 consecutive patients with degenerative mitral valve disease were included in the study. All had quadrangular resection of a P2 prolapse and annuloplasty with a Cosgrove-Edwards annuloplasty band. Clinical and echocardiography follow-up was complete.

## Results

There was no intraoperative or 30 day mortality. After a mean follow-up of 5.0 ± 1.9 years, survival was 94.3%. At midterm, freedom from reoperations was 98.9%, freedom from thromboembolism was 97.5% and freedom from endocarditis was 99.4%. Echocardiography follow-up showed recurrent mitral valve regurgitation higher than grade 2 in two patients. Mean ejection fraction was 60.3 ± 10.2%, left atrial diameter was 42 ± 7 mm, mean gradient was 3.2 ± 1.4 mmHg, effective orifice area was 3.3 ± 1.3cm², mitral leaflet coaptation length was 7.5 ± 1.9 mm and mitral leaflet tethering height was 6.2 ± 2.3 mm.

## Conclusion

Mitral valve repair using the Cosgrove annuloplasty band for degenerative mitral valve disease provides an effective and durable form of reconstruction.

